# Perception of Indian Dental Surgeons regarding Molar Incisor Hypomineralization

**DOI:** 10.5005/jp-journals-10005-1496

**Published:** 2018-04-01

**Authors:** Sumita Upadhyay, Gyanendra Kumar, Jatinder K Dhillon, Namrata C Gill

**Affiliations:** 1 Assistant Professor, Department of Pediatric Dentistry, Kathmandu University School of Medical Sciences, Dhulikhel Hospital, Dhulikhel, Nepal; 2 Associate Professor, Department of Pedodontics and Preventive Dentistry, Maulana Azad Institute of Dental Sciences, New Delhi, India; 3 Assistant Professor, Department of Pedodontics and Preventive Dentistry, Maulana Azad Institute of Dental Sciences, New Delhi, India; 4 Assistant Professor, Department of Pedodontics and Preventive Dentistry Dr. Harvansh Singh Judge Institute of Dental Sciences & Hospital, Panjab University, Chandigarh, India

**Keywords:** Dental surgeons, Molar incisor hypomineraliza-tion, Perception.

## Abstract

**Aim:**

To determine the perception of Indian dental professionals about prevalence, severity, and etiological factors of molar incisor hypomineralization (MIH).

**Materials and methods:**

An online survey was mailed to dental professionals encompassing various questions regarding etiology, diagnosis, prevalence, and management of MIH.

**Results:**

More than 90% of the respondents encounter teeth with hypomineralization in their practice, with less than half of them encountering such teeth on a monthly basis. Among these more than one-third find it difficult to manage one or the other aspect of MIH. The etiology was found to be varying as per the respondents.

**Conclusion:**

Molar incisor hypomineralization is a common condition encountered by dental professionals with no apparent consensus regarding the anticipated prevalence, severity, and etiology of this condition. Knowledge of clinicians’ level of perception could be an incentive for pediatric dentists to become more acquainted with MIH by conducting research into its different aspects.

**How to cite this article:** Upadhyay S, Kumar G, Dhillon JK, Gill NC. Perception of Indian Dental Surgeons regarding Molar Incisor Hypomineralization. Int J Clin Pediatr Dent 2018;11(2):116-121.

## INTRODUCTION

Incisors and first permanent molars with severe hypomineralized enamel of unknown etiology were first acknowledged in Sweden in the late 1970s.^1^ This condition has been referred to as “idiopathic enamel hypomineralization in permanent first molars,” “hypo-mineralized permanent first molars,” “cheese molars,” and “nonfluoride hypomineralization in permanent first molars.”^1-6^ The term ”MIH” was introduced by Weerheijm et al^7^ to describe this condition. Molar incisor hypomineralization is defined as hypomineralization of systemic origin of permanent first molars, frequently associated with affected incisors. One or more of the molars may be affected, each with different degrees of severity. The permanent incisors may also be affected. Even though MIH is defined as a chronological and general disturbance, the number of permanent first molars and the degree of hypomineralization vary extensively.

The clinical management of MIH is challenging for the dentist due to sensitivity and rapid development of dental caries in affected permanent first molars, difficulty in achieving anesthesia, unpredictable behavior of apparently intact opacities, and repeated marginal breakdown of restorations and difficulty in cavity preparation.^8,9^ This is due to enamel in teeth affected by MIH exhibiting disorganized enamel prisms, a porous structure, and loosely packed crystallites.^10-14^

The prevalence varies considerably from 2.8% in Hong Kong Chinese children^15^ to almost 40% in Denmark and Brazil.^16,17^ The reasons for this variation may be lack of consistent and standardized assessment criteria. There have been recent surveys among pediatric dentists in Europe,^18^ Australia and New Zealand,^19^ and Iraq^20^ to ascertain the knowledge and practice regarding MIH. Such surveys aim to reduce the ambiguity regarding this unique clinical condition and also to shed light on various aspects of MIH in terms of management strategies.

Despite the clinical significance of MIH, little information is available on the perception of Indian dental surgeons regarding MIH in the country. There have been few prevalence studies in India.

The aim of this survey was to assess the experience of Indian dental surgeons regarding prevalence, clinical problems, and management of MIH with the broader aim of highlighting the need for prevalence studies for MIH in India.

## MATERIALS AND METHODS

The study was conducted between November 2017 and January 2018 after taking approval from ethical committee of Maulana Azad Institute of Dental Sciences, New Delhi, India.

A questionnaire was modeled on the ones by Weerheijm and Mejare^18^ and Ghanim et al^20^ after obtaining permission from the authors. The clinical pictures used in the study were the same as those used by Weerheijm and Mejare.^18^ There were a total of 15 multiple choice questions (Table 1). This questionnaire was then sent out to 1,500 Indian dental surgeons throughout the country including pediatric dentists using Google surveys. The data were compiled using MS Excel 2007 (Microsoft) and analyzed. Three reminders were sent at monthly intervals.

**Table 1:** **Table 1:** Questionnaire

1		Educational qualification		Bachelor of dental surgery Master of dental surgery	
2		Specialty		Pediatric dentist Nonpediatric dentist	
3		This question relates to the above image. Do you encounter such teeth in your practice? (image shown in the Google survey)		Yes No	
4		In your clinical work how often do you notice hypomineralized teeth?		Often in a week Occasionally in a week Weekly Monthly Yearly	
5		Regarding the severity of defect, which of the following do you most frequently notice in your practice? (select all that apply)		White demarcation alone Yellow/brown demarcation alone White + yellow/brown demarcation Yellow/brown demarcation + PEB White demarcation + PEB All three together Did not notice	
6		In your practice do you feel the incidence of hypomineralized teeth has increased over the last 10 years, or in the period of your practice (if less than 10 years)?		Yes No	
7		Approximately what percentage of patients do you observe these teeth in?		Less than 5% 5-10% 10-25% More than 25%	
8		How frequently do you notice this defect in the second primary molar tooth in comparison to first permanent molar?		More frequently Equal Less frequently	
9		Which factors do you think are involved in the etiology of MIH? (select all that apply)		Genetic Medications taken by mother during pregnancy Chronic medical condition affecting the mother during pregnancy Acute medical conditionaffecting the mother during pregnancy Fluorides Environmental contaminants Chronic medical condition affecting the involved child Acute medical condition affecting the involved child Medication taken by the involved child Other	
10		Do you think MIH is challenging to manage?		Yes, very difficult Yes, somewhat difficult No	
11		If yes to the previous question, do you experience problems with? (select all that apply)		Diagnosis Esthetics Achieving adequate local anesthesia Determining margins of affected tooth Providing adequate restoration Long-term success of restoration Achieving patient comfort All of the above	
12		Which factors influence your choice of restorative material in such cases? (select all that apply)		Adhesion Esthetics Patient or parent preference Durability Remineralization potential Sensitivity Personal experience Research findings Others	
13		Which material do you use to restore these teeth? (select all that apply)		High-fluoride GIC GIC Resin-modified GIC Compomer Composite Flowable composite Amalgam Preformed crowns Cast restorations Others	
14		Would you like to seek more information regarding tooth hypomineralization?		Yes No	
15		In which part/parts do you think you need further information? (select all that apply)		Etiology Diagnosis Management

## RESULTS

**Graph 1:**
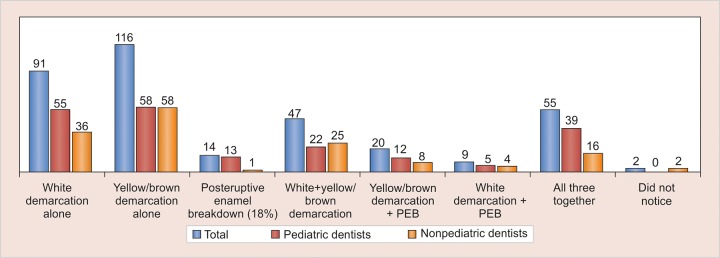
Clinical presentation observed by dental clinicians in MIH

Of the 1,500 questionnaires mailed, 393 responses were received, with a response rate of 26.2%. Twenty-eight questionnaires were partially completed. Responses were received from various clinicians as well as academicians and postgraduate students, which included 217 pediatric dentists and 176 nonpediatric dentists. It was observed that 96.3% of the total respondents encounter such teeth. Regarding the frequency, 43% of the pediatric dentists encounter such teeth often in a week, whereas overall 41.3% respondents reported that the frequency is monthly. The most commonly encountered clinical appearance was yellow/brown demarcation alone or associated with white demarcation and/or posteruptive breakdown (44%), whereas only 3.56% (14 respondents) reported observing only posteruptive enamel breakdown (PEB) (Graph 1).

About 42% respondents reported that the incidence of hypomineralized teeth has increased. Another interesting finding was that there was a lot of variation among the respondents regarding the prevalence of such teeth in patients with 52% pediatric dentists reporting that 5 to 10% of their patients report with such teeth and 57% other respondents reporting it to be less than 5%. There was general consensus among majority of respondents (78.7%) that such condition is more frequently seen in permanent first molars rather than deciduous second molars. There was significant discrepancy among pediatric dentists and other respondents regarding the rise in incidence; 53% pediatric dentists and 27% other respondents reported an increase in the incidence.

**Graph 2:**
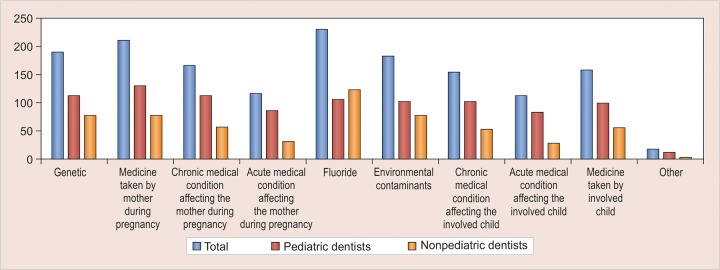
Distribution of responses regarding etiology of MIH

**Graph 3:**
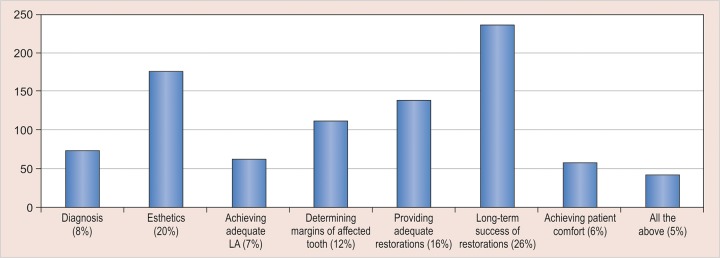
Challenges faced by dental clinicians regarding various aspects of MIH

**Graph 4:**
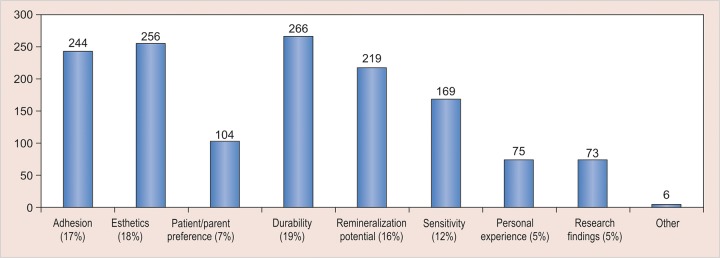
Factors under consideration in selection of restoration in teeth affected by MIH

**Graph 5:**
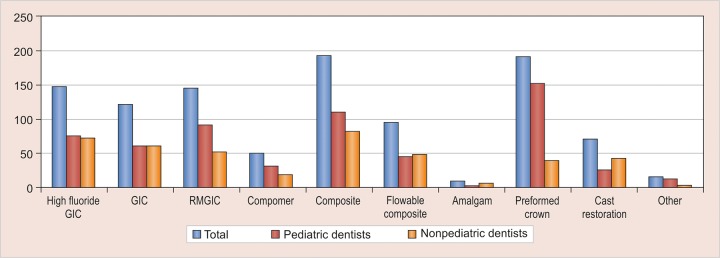
Restorative materials used for MIH

**Graph 6:**
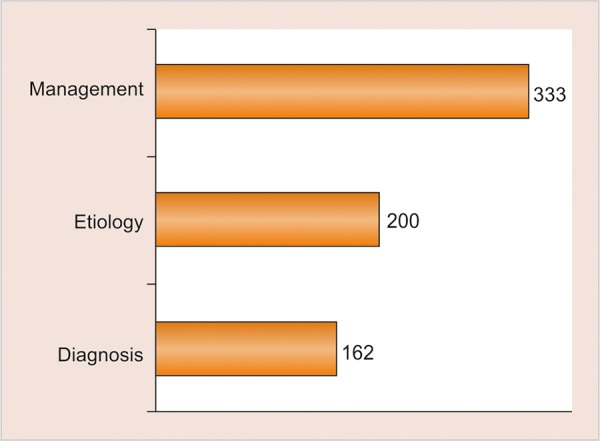
Areas perceived in which knowledge is required regarding MIH

There was no agreement among the respondents regarding the etiology of MIH and 15% of the respondents attributed it to fluorides (Graph 2); 88.3% respondents reported that they found MIH to be challenging to manage and most of them faced problems ranging from diagnosis, clinical management, and prognosis (Graph 3). Durability and esthetics were considered to be the major factors influencing choice of restorative material followed by adhesion and remineralization potential (Graph 4). There was a wide variation in the choice of restorative material in such teeth with pediatric dentists veering toward preformed crowns (Graph 5). About 88.5% respondents felt that they require more information regarding management of MIH (Graph 6).

## DISCUSSION

The epidemiology of dental caries and periodontal diseases has been widely studied in India. However, literature on MIH is sparse, although it is recognized as a distinct clinical entity in India. This is probably the first study investigating perception of Indian dentists regarding this condition. The majority of participants in this study had encountered teeth affected by MIH, which is in agreement with the results of previous studies conducted in Europe,^18^ Australia and New Zealand,^19^ and Iraq.^20^

In the present study, it was observed that pediatric dentists encounter such teeth more frequently than other dental practitioners. This may be attributed to the fact that pediatric dentists encounter children exclusively as compared with any other dental practitioner. Also adult patients may often have had restoration of such teeth or the affected tooth/teeth might have been lost and no longer exhibit signs of MIH when reporting to the dentist thus leading to the lower prevalence reported. Another factor responsible for such a skewed response might be that other practitioners often lack knowledge about this condition so they might be missing out on the presentation. The fact holds true that eyes see only what the mind knows. There was a varied response regarding prevalence of MIH with 52% pediatric dentists reporting the prevalence to be 5 to 10%, while most of others (57%) reporting it to be less than 5%. This disparity could be attributed to various reasons, such as different birth cohorts or different ages of the children at examination. It could also reflect real differences between regions. There may have been differences in criteria for examination of these teeth. This emphasizes the need to perform a survey of this condition with calibrated examiners. Moreover, due to varied presentation depending upon the extent of involvement, age of patient at the time of diagnosis, and degree of damage, it becomes difficult to categorize MIH. The most common clinical manifestation encountered by the participants in our study was yellow/brown demarcation either alone or in combination with others (44%) and the least commonly seen was PEB (18%). This might be explained by the fact that yellow/brown enamel opacities have shown to be more porous than lighter opacities.^21^ A correlation between hardness values, mineral density, and the color of the hypomineralized enamel has been shown, with yellow/brown opacities being softer than white.^22,23^ This leads to early breakdown and associated symptoms causing the affected patients to seek treatment. Yellow/brown opacities are easily distinguishable from fluorosis and white spot lesions due to caries as compared with other clinical manifestations of MIH leading to early differentiation. Moreover, PEB may often go unrecognized. Posteruptive enamel breakdown most often occurs shortly after eruption, when the affected tooth is under occlusal load.^24^ This may be mistaken as dental caries by the untrained eye.

Scientists and clinicians are still trying to establish the exact etiological factors for MIH. There is still plenty of ambiguity regarding the etiology of MIH. Several factors have been proposed but there is no consensus as all the studies have been retrospective in nature. This ambiguity is reflected in the response of the participants as well. There is a need to have experimental, animal, and prospective cohort studies on MIH to establish the exact etiology.

Molar incisor hypomineralization is a unique condition associated with difficulty in diagnosis due to lack of a uniform assessment and identification criteria. Patients affected with MIH are more likely to have behavior management problems as well as dental fear and anxiety due to repeated treatments and hypersensitivity.^8^ This is reflected in the responses of the present survey.

There is frequent breakdown of restorations due to the porous nature of enamel in MIH, thus making durability of the restoration a critical factor as evidenced by the participant’s responses [etching and bonding difficulties, adhesion failures with glass ionomer cements (GICs) due to improper crystal lattice]. Another interesting finding of this survey was that pediatric dentists preferred preformed crowns as the restoration of choice as compared with other dental surgeons. This might be attributed to familiarity with use of preformed crowns among the former (multisurface involvement, superadded with dental decay).

A large majority of participants expressed an interest in obtaining further information about MIH, thus highlighting that there is awareness regarding this condition among Indian dental surgeons.

Limitations of the study:

- There is a low response rate of mailed questionnaires due to lack of personal contact. There is no opportunity to cross examine the responses.

## CONCLUSION

Molar incisor hypomineralization is a common condition encountered by dental professionals with no apparent consensus regarding the anticipated prevalence, severity, and etiology of this condition. Thus, there is need for conducting research in various aspects of MIH.

### What This Article Adds

Molar incisor hypomineralization is commonly encountered by Indian dental clinicians and is considered to be a common clinical problem.

### Why This Article is Important for Pediatric Dentists

- The results of this survey will raise awareness about this unique and difficult to manage clinical condition.
- This survey might motivate others to look into research on various aspects of MIH.
